# Glucosylceramide synthase upregulates *MDR1 *expression in the regulation of cancer drug resistance through *cSrc *and β-catenin signaling

**DOI:** 10.1186/1476-4598-9-145

**Published:** 2010-06-11

**Authors:** Yong-Yu Liu, Vineet Gupta, Gauri A Patwardhan, Kaustubh Bhinge, Yunfeng Zhao, Jianxiong Bao, Harihara Mehendale, Myles C Cabot, Yu-Teh Li, S Michal Jazwinski

**Affiliations:** 1 Department of Basic Pharmaceutical Sciences, University of Louisiana at Monroe, Monroe, Louisiana 71209, USA; 2 Department of Pharmacology, Toxicology and Neuroscience, Louisiana State University Health Sciences Center, Shreveport, Louisiana 71130, USA; 3 Department of Pathology, Louisiana State University Health Sciences Center, Shreveport, Louisiana 71130, USA; 4 Department of Toxicology, University of Louisiana at Monroe, Monroe, Louisiana 71209, USA; 5 Department of Experimental Therapeutics, John Wayne Cancer Institute, Santa Monica, California 90404, USA; 6 Department of Biochemistry, Tulane University School of Medicine, New Orleans, Louisiana 70112, USA; 7 Department of Medicine and Tulane Center for Aging, Tulane University School of Medicine, New Orleans, Louisiana 70112, USA

## Abstract

**Background:**

Drug resistance is the outcome of multiple-gene interactions in cancer cells under stress of anticancer agents. *MDR1 *overexpression is most commonly detected in drug-resistant cancers and accompanied with other gene alterations including enhanced glucosylceramide synthase (GCS). *MDR1 *encodes for P-glycoprotein that extrudes anticancer drugs. Polymorphisms of *MDR1 *disrupt the effects of P-glycoprotein antagonists and limit the success of drug resistance reversal in clinical trials. GCS converts ceramide to glucosylceramide, reducing the impact of ceramide-induced apoptosis and increasing glycosphingolipid (GSL) synthesis. Understanding the molecular mechanisms underlying *MDR1 *overexpression and how it interacts with GCS may find effective approaches to reverse drug resistance.

**Results:**

*MDR1 *and *GCS *were coincidently overexpressed in drug-resistant breast, ovary, cervical and colon cancer cells; silencing *GCS *using a novel mixed-backbone oligonucleotide (MBO-asGCS) sensitized these four drug-resistant cell lines to doxorubicin. This sensitization was correlated with the decreased *MDR1 *expression and the increased doxorubicin accumulation. Doxorubicin treatment induced GCS and *MDR1 *expression in tumors, but MBO-asGCS treatment eliminated "in-vivo" growth of drug-resistant tumor (NCI/ADR-RES). MBO-asGCS suppressed the expression of *MDR1 *with GCS and sensitized NCI/ADR-RES tumor to doxorubicin. The expression of P-glycoprotein and the function of its drug efflux of tumors were decreased by 4 and 8 times after MBO-asGCS treatment, even though this treatment did not have a significant effect on P-glycoprotein in normal small intestine. GCS transient transfection induced *MDR1 *overexpression and increased P-glycoprotein efflux in dose-dependent fashion in OVCAR-8 cancer cells. GSL profiling, silencing of globotriaosylceramide synthase and assessment of signaling pathway indicated that GCS transfection significantly increased globo series GSLs (globotriaosylceramide Gb3, globotetraosylceramide Gb4) on GSL-enriched microdomain (GEM), activated cSrc kinase, decreased β-catenin phosphorylation, and increased nuclear β-catenin. These consequently increased *MDR1 *promoter activation and its expression. Conversely, MBO-asGCS treatments decreased globo series GSLs (Gb3, Gb4), cSrc kinase and nuclear β-catenin, and suppressed *MDR-1 *expression in dose-dependent pattern.

**Conclusion:**

This study demonstrates, for the first time, that GCS upregulates *MDR1 *expression modulating drug resistance of cancer. GSLs, in particular globo series GSLs mediate gene expression of *MDR1 *through cSrc and β-catenin signaling pathway.

## Background

Chemotherapy is the principal treatment option for patients with late stage cancers. Despite considerable advances in drug discovery, metastatic solid malignancies remain incurable, due to their poor response to most of the conventional antineoplastic agents. Acquired drug resistance of cancer cells severely limits the success of chemotherapy, particular in solid tumors [1,2]. The ABCB1 transporter, known as P-glycoprotein (P-gp) is encoded by human multidrug resistance 1 gene (*MDR1*) and is an important mediator of drug resistance [2,3]. Like other membrane transport proteins in ABC (ATP binding cassette) family, P-gp is found in various cellular membranes of organisms from bacteria to mammals. P-gp plays roles in the absorption, distribution, and excretion of pharmacological compounds in normal tissues [4,5]. However, overexpression of *MDR1 *in tumors results in increase of P-gp and active effluxing of a variety of natural product anticancer agents from cells [2,6]. The polymorphism of *MDR1*, particularly the 'silent' polymorphism, blocks the effects of currently available P-gp antagonists and thus limits the success of these agents in clinical trials [7-10].

Drug resistance is the outcome of multiple-gene interactions in cancer cells under the stress of antineoplastic agents. Several drug-resistant markers including Bcl-2, mutant p53, and glucosylceramide synthase (GCS) are overexpressed in drug-resistant cancers [5,11-13]. However, little is known about the molecular mechanism underlying *MDR1 *overexpression and how it interacts with other genes to impart drug-resistance. Recently, an emerging body of evidence indicates a curious association of multidrug resistance with ceramide glycosylation [13-18]. GCS (UDP-glucose:ceramide glucosyltransferase, *UGCG*) transfers a glucose residue from UDP-glucose to ceramide and produces glucosylceramide [19,20]. This first step in glycosphingolipid (GSL) synthesis tightly regulates the production of all upstream GSLs [21]. Ceramide, a lipid second messenger, induces growth arrest or apoptosis in cancer cells; this induced-apoptosis is in part responsible for the therapeutic efficiency of antineoplastic regimens including anthracyclines, taxanes, and *vinca *alkaloids and radiation therapy [15,22-25]. Overexpression of GCS can result in drug resistance, as introduction of GCS confers cell resistance to doxorubicin, daunorubicin, and tumor necrosis factor-α [16,26,27]. GCS is overexpressed in many MDR cancer cell lines [17,28], and in leukemia, breast cancer, and renal cell cancer [29-31]. Interestingly, GCS is coincidently overexpressed with *MDR1 *in drug-resistant cells [28,32] and in leukemia cells from patients who have poor-response to chemotherapy [31,33]. We have studied the effects of ceramide glycosylation on *MDR1 *and found that GCS upregulates *MDR1 *expression through activation of cSrc and β-catenin signaling.

## Results

### Silencing GCS represses *MDR1 *expression and sensitizes cancer cells to chemotherapeutic agents

**Figure 1:**
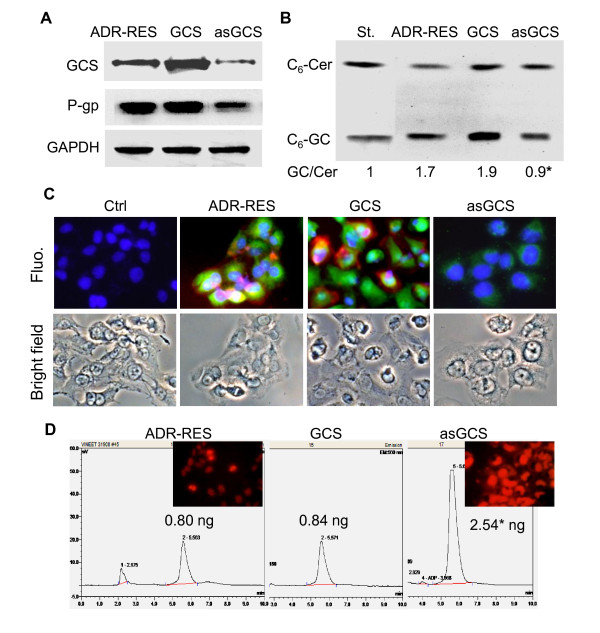
**Effects of GCS on P-gp in NCI/ADR-RES Transfectants**. (A) GCS and P-gp proteins detected by Western blot. Detergent-soluble protein (50 μg/lane) from NCI/ADR-RES (ADR-RES), NCI-ADR-RES/GCS (GCS) and NCI/ADR-RES/asGCS (asGCS) cells was immunoblotted with anti-GCS or anti-P-gp antibody. GAPDH was used as loading control. (B) Ceramide glycosylation catalyzed by GCS. Cells were incubated with NBD C6-Cer (100 nM) in 1% BSA RPMI-1640 medium, at 37°C for 2 hr. C6-Cer and C6-GlcCer were identified on chromatograms with commercial standard (St.) and measured using spectrophotometry. *, p < 0.001 compared to ADR-RES cells. (C) Immunostaining of GCS and P-gp. Cells were incubated with anti-human GCS (green) and anti-P-gp (red) following addition of Alexa 488- and Alexa 667-conjugated secondary antibodies. DAPI in mounting solution was used for nucleus counterstaining (blue). Ctrl, NCI/ADR-RES cells were incubated with the secondary antibodies alone, as specificity control; Fluo, merged fluorescence microphotograph (× 200). (D) Doxorubicin accumulation. After 1 hr incubation with doxorubicin (0.1 mg/ml), cellular doxorubicin was documented by fluorescence microscopy (× 200) and analyzed by HPLC, following methanol extraction. Doxorubicin amount was normalized to 100,000 cells. *, p < 0.001.

We observed the role of GCS in the regulation of *MDR1 *expression in NCI/ADR-RES and its GCS transfectants. GCS protein levels were increased in NCI/ADR-RES/GCS cells and significantly decreased in NCI/ADR-RES/asGCS cells (Figure 1A). Consistent with these, GCS enzyme activity was decreased to 52% (0.9 *vs*. 1.7 GC/Cer) in NCI/ADR-RES/asGCS cells, whereas the activity was increased to 110% (1.9 *vs*. 1.7 GC/Cer) in NCI/ADR-RES/GCS, as compared with parental NCI/ADR-RES cells (Figure 1A,B). Coordinately, P-gp was significantly decreased to 40% in NCI/ADR-RES/asGCS cells, as detected by Western blotting and immunostaining (Figure 1A,C). HPLC assays indicated that cellular accumulation of doxorubicin was increased by 3-fold in NCI/ADR-RES/asGCS cells (2.54 ± 0.15 *vs*. 0.80 ± 0.51 ng/10^5 ^cells) as compared with NCI/ADR-RES cells (Figure 1D). Substantial intracellular accumulation of doxorubicin was also observed in NCI/ADR-RES/asGCS cells under fluorescence microscopy (Figure 1D).

**Figure 2:**
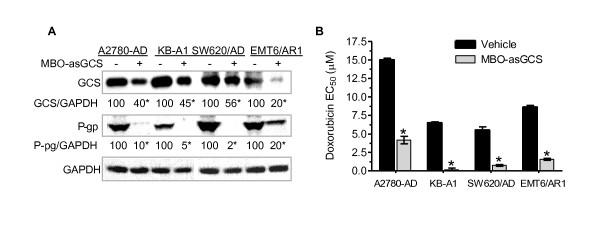
**Silencing of GCS by MBO-asGCS Represses *MDR1 *Expression and Sensitizes Drug-Resistant Cancer Cells**. (A) P-gp and GCS in drug-resistant cancer cells. Cells were cultured in growth medium for 24 hr, and then treated with vehicle or MBO-asGCS (50 nM) for an additional 48 hr. Equal amounts of protein (50 μg/lane) were resolved by 4-20% gradient SDS-PAGE and immunoblotted with anti-GCS and anti-P-gp antibodies. GAPDH was used as endpoint control, and GCS/GAPDH or P-gp/GAPDH represents optical densities of the bands. -, vehicle (Lipofectamine 2000); +, MBO-asGCS (50 nM). *, p < 0.001 compared with vehicle treatment. (B) Cell response to doxorubicin. After pretreatment of MBO-asGCS (50 nM) or vehicle, cells were incubated with 5% FBS medium at the presence of doxorubicin for an additional 72 hr. *, p < 0.001 compared with vehicle.

*MDR1 *and GCS have been shown to be coincidently overexpressed in several drug-resistant cell lines [17,28]. To validate the association of *MDR1 *expression with GCS, we assessed *MDR1 *expression in four different types of cancer cells, in the absence and presence of GCS silencing with MBO-asGCS (a mixed-backbone oligonucleotide against human GCS) [34,35]. Results showed that GCS protein levels were decreased to 40%, 45%, 56% and 20% of control, respectively in drug-resistant human A2780-AD ovary cancer, KB-A1 cervical cancer, SW620/AD colon cancer, and murine EMT6/AR1 breast cancer cells treated with MBO-asGCS (50 nM, 7 days) (Figure 2A). Consistently, MBO-asGCS treatments decreased P-gp protein levels to 10%, 5%, 2% and 20% of control in these cell lines. Furthermore, MBO-asGCS substantially sensitized cells to doxorubicin; the EC_50 _values for doxorubicin were decreased by 4-fold in A-2780AD, 43-fold in KB-A1, 7-fold in SW620/AD, and 6-fold in EMT6/AR1 cells, respectively (Figure 2B). These data indicate that suppressing GCS can repress *MDR1 *expression and reverse cellular resistance to anticancer agents.

### Silencing GCS represses *MDR1 *expression and restores tumor sensitivity to doxorubicin

**Figure 3:**
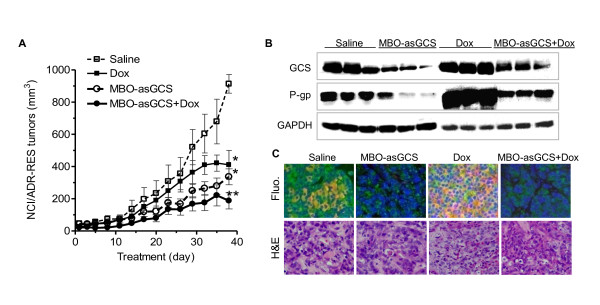
**Silencing of GCS by MBO-asGCS Represses *MDR1 *Expression and Reverses Tumor Resistance to Doxorubicin *in vivo***. (A) Tumor growth. Tumors generated from NCI/ADR-RES cells (~3 mm in diameter, 10 mice/group) were treated with MBO-asGCS (1 mg/kg every 3 days) or doxorubicin (2 mg/kg/week) and combination thereof. Data represent the mean ± SE; *, p < 0.001 compared to saline group (open squares); **, p < 0.001 compared to doxorubicin treatment (solid squares). (B) Western blots of tumor tissues. After treatments, extracted tumor proteins (100 μg/μl, three samples per group) were resolved by 4-20% SDS-PAGE and immunoblotted with anti-GCS or anti-P-gp antibodies, respectively. (C) Immunostaining. After retrieval, antigens on tissue sections (5 μm) were recognized by anti-GCS (green) and anti-P-gp (red) antibodies with fluorescence conjugated secondary antibodies. Microphotographs of merged fluorescence (Fluo.) with H&E staining (H&E) were originally magnified by 200.

Inoculation of NCI/ADR-RES cells into athymic mice generated MDR tumor xenografts. Mice with MDR xenografts were treated with MBO-asGCS (1 mg/kg per 3 days) alone or combined with doxorubicin (2 mg/kg per week). As shown in Figure 3A, MBO-asGCS treatment significantly decreased tumor growth and increased the sensitivity of these tumors to doxorubicin. The combination treatment (MBO-asGCS + Dox) decreased tumor volumes to 45% (187 *vs*. 411 mm^3^, p < 0.01) and 20% (187 *vs*. 913 mm^3^, p < 0.001), respectively, as compared with doxorubicin or saline treatment. Western blot analysis revealed that GCS and P-gp protein levels were increased 2-fold and 4-fold in tumors treated with doxorubicin, as compared with saline (Figure 3B). However, MBO-asGCS decreased both GCS and P-gp protein levels by approximately 4-fold in tumors treated with MBO-asGCS alone or MBO-asGCS combined with doxorubicin, as compared with saline and doxorubicin groups (Figure 3B). These findings were confirmed in tumor tissues after immunostaining for GCS and P-gp (Figure 3C).

**Figure 4:**
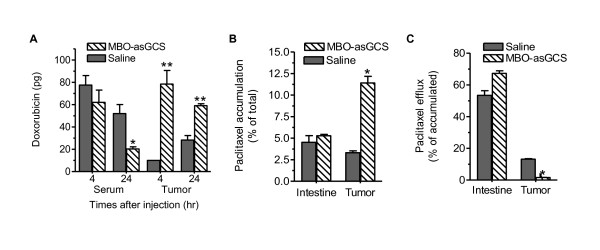
**Effects of GCS Silencing on P-gp Regulated Drug Accumulation and Efflux in Tumors**. NCI/ADR-RES tumors were treated with MBO-asGCS (1 mg/kg/3 days, 3 mice/group) or saline for 7 days. (A) Doxorubicin accumulation. After 4 hr and 24 hr peritoneal administration of doxorubicin (1 mg/kg), serum and tumor tissues were collected and prepared for HPLC assays. Doxorubicin levels were represented per μl of serum or per mg of tumor tissue. *, p < 0.001 compared with serum of saline treatment at 24 hr; **, P < 0.001 compared with saline treatments. (B) Paclitaxel accumulation. After two administrations of MBO-asGCS, tissue suspensions (25 mg/reaction) were incubated with Flutax-2 (1 μM) in medium containing collagenase IV, immediately following mincing. Accumulation of paclitaxel was measured after 2 hr incubation. *, p < 0.001 compared with saline treatment of tumors. (C) Paclitaxel efflux. After accumulation described in (B), tissues were incubated with fresh medium for an additional 2 hr to measure paclitaxel efflux. *, p < 0.001 compared with saline treatment of tumors.

In agreement with its repressive effect on P-gp protein, MBO-asGCS significantly increased doxorubicin accumulation in tumor tissues. As shown in Figure 4A, MBO-asSGCS treatment augmented tumor accumulation of doxorubicin by 8-fold (78 pg/mg *vs*. 10 pg/mg) and 2-fold (59 pg/mg *vs*. 28 pg/mg) after 4 and 24 hrs of doxorubicin administration, compared to the saline group, respectively. Meanwhile, serum levels of doxorubicin decreased in the MBO-asGCS group. Further assessment using Flutax-2 (Oregon green 488-paclitaxel, a substrate of the P-gp pump) revealed that paclitaxel accumulation was increased 4-fold (11.41% *vs*. 3.32% of total, p < 0.001, Figure 4B), since that paclitaxel efflux decreased by 8-fold (1.66% *vs*. 13.2% of accumulated, p < 0.001) in tumors treated with MBO-asGCS (Figure 4C). MBO-asGCS treatments did not have a significant effect on P-gp of normal small intestine. These results indicate that silencing GCS by MBO-asGCS efficiently represses *MDR1 *expression and reverses *in vivo *drug resistance.

### GCS upregulates *MDR1 *expression through cSrc kinase and β-catenin signaling

**Figure 5:**
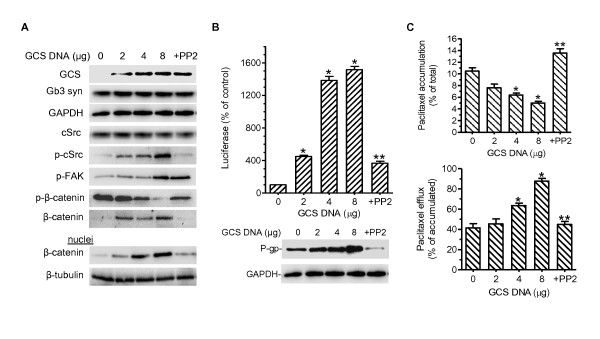
**GCS Upregulates *MDR1 *Expression through Enhanced cSrc/β-Catenin Signaling**. After a series of transient GCS transfection (0, 2.0, 4.0, 8.0 μg of pcDNA 3.1-GCS plasmid DNA in 100-mm dish), OVCAR-8 cells were cultured in 10% FBS RPMI-1640 medium for 7 days. OVCAR-8 cells transfected with pcDNA 3.1-GCS (8 μg) were then treated with 10 μM PP2 for 24 hr (+PP2). (A) Western blots. Equal amounts of detergent-soluble total cellular proteins or nuclear proteins (50 μg/lane) were resolved on 4-20% gradient SDS-PAGE and immunoblotted with indicated primary antibodies. Gb3 syn, Gb3 synthase; p-cSrc, phosphorylated cSrc; p-FAK, phosphorylated FAK; p-β-catenin, phosphorylated β-catenin. (B) *MDR1 *expression. *MDR1 *promoter activity (top panel) and P-gp protein (bottom panel) were assessed as described in Methods, after 7 days of GCS transient transfection in OVCAR-8 cells. *, p < 0.001 compared with mock transfection; **, p < 0.001 compared with vehicle treatment in cells transfected with GCS (8 μg DNA). (C) Paclitaxel accumulation and efflux. Cells were incubated with Flutax‐2 (0.5 μM) in medium at 37°C. Accumulation of paclitaxel (top panel) was measured after 2 hr incubation. After washing with ice-cold PBS, cells were re-incubated with fresh medium for an additional 2 hr to measure efflux (bottom panel). *, p < 0.001 compared with the mock transfection. **, p < 0.001 compared with vehicle treatment in cells transfected with GCS (8 μg DNA).

We transiently transfected GCS in human OVCAR-8 ovarian carcinoma cells, which express low level of *MDR1 *[36], in order to explore putative mechanisms underlying GCS upregulation of *MDR1*. One week after the transfection, GCS protein levels were elevated by 10-fold, 20-fold and 25-fold in cells transfected with increasing amounts of GCS plasmid DNA (2, 4, and 8 μg/dish) (Figure 5A). Interestingly, the levels of phosphorylated cSrc and FAK proteins were enhanced corresponding to GCS levels, even though the total cSrc levels remained relatively unchanged. Nuclear β-catenin levels were elevated by 5- to 10-fold whereas phosphorylated β-catenin declined as GCS, p-cSrc, and p-FAK increased in these cells (at 4, and 8 μg/dish). P-gp protein levels were elevated by 2 to 3-fold concomitant with *MDR1 *promoter activities in these cells (4 and 8 μg/dish) (Figure 5B). The pyrazolo pyrimidine (PP2), a Src kinase inhibitor [37,38] selectively decreased the levels of phosphorylated cSrc (not p-FAK) and increased phosphorylated β-catenin, but did not decrease Gb3 synthase of cells after GCS transfection (8 mg/dish, Figure 5A). The PP2 treatments blocked the stimulation effect of GCS on nuclei β-catenin, further *MDR1 *promoter activity and P-gp expression (Figure 5A,B). We also assessed the cellular accumulation and activity of P-gp using Flutax-2. As shown in Figure 5C, paclitaxel accumulation was decreased to 60% and 47% of control in cells transfected with increasing amounts of GCS plasmid (4, 8 μg DNA/dish), respectively, as compared with mock transfection. Conversely, cell efflux of paclitaxel was elevated to 153% and 212% over control in these transfectants. Inhibition of cSrc kinase by PP2 treatment eliminated the effects of GCS transfection on P-gp activity in the accumulation and efflux of paclitaxel (Figure 5C).

### Silencing GCS represses *MDR1 *transactivation via inhibition of cSrc and β-catenin signaling

**Figure 6:**
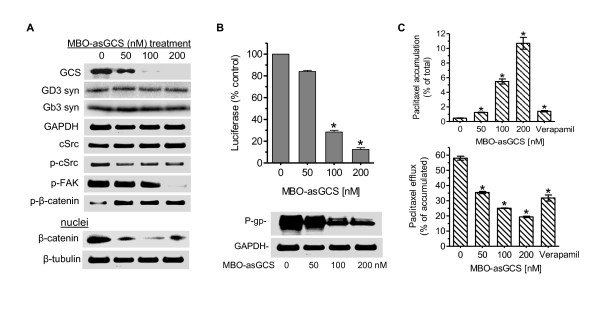
**Silencing GCS Represses *MDR1 *Expression by Decreasing cSrc/β-Catenin Signaling**. After MBO-asGCS treatments (0, 50, 100, 200 nM), drug resistant NCI/ADR-RES cells were cultured in 10% FBS RPMI-1640 medium for 7 days. The NCI/ADR-RES cells were incubated with verapamil (10 μg, 2 hr) in 5% FBS RPMI-1640 medium to inhibit P-gp function. (A) Western blots. Equal amounts of total cellular proteins or nuclear proteins (50 μg/lane) were resolved by 4-20% gradient SDS-PAGE and immunoblotted with indicated primary antibodies. GD3 syn, GD3 synthase; Gb3 syn, Gb3 synthase; p-cSrc, phosphorylated cSrc; p-FAK, phosphorylated FAK; p-β-catenin, phosphorylated β-catenin. (B) *MDR1 *expression. *MDR1 *promoter activity (top panel) and P-gp protein (bottom panel) were assessed as described in Methods, after 7 days of MBO-asGCS treatments. *, p < 0.001 compared with vehicle. (C) Paclitaxel accumulation and efflux. Cells were incubated with Flutax-2 (0.5 μM) in medium at 37°C for 2 hr to measure paclitaxel accumulation (top panel). After washing with ice-cold PBS, cells were incubated with fresh medium for an additional 2 hr to measure paclitaxel efflux (bottom panel). *, p < 0.001 compared with vehicle treatment.

We silenced GCS with MBO-asGCS in NCI/ADR-RES cells that overexpressed GCS and *MDR1*, to verify the mechanism underlying GCS modulation of *MDR1*. After one week of MBO-asGCS treatments, GCS protein levels, but neither GD3 synthase nor Gb3 synthase, were decreased to 30%, 5% and 2% in cells treated with increasing concentrations of MBO-asGCS (50, 100, 200 nM), as compared with vehicle control, respectively (Figure 6A). The levels of the phosphorylated cSrc and FAK proteins, but not the total cSrc, were correspondingly decreased with GCS protein levels. Interestingly, nuclear β-catenin was decreased to approximately 10%, whereas phosphorylated β-catenin was increased as the levels of GCS, p-cSrc, and p-FAK were decreased in these cells after MBO-asGCS treatments. P-gp protein levels were decreased to 85%, 30% and 25% with decreases in *MDR1 *promoter activity of cells treated with increasing concentrations of MBO-asGCS (50, 100, 200 nM) (Figure 6B). Sequentially, we found that cellular accumulation of paclitaxel was elevated by 2.5-fold, 11-fold and 22-fold in cells treated with MBO-asGCS, compared to vehicle control, respectively (Figure 6C). Conversely, cell efflux of paclitaxel was reduced to 89%, 48%, and 31% in cell after these treatments. As anticipated, verapamil treatment (10 μM) inhibited P-gp function, as effectively as MBO-asGCS (50 nM) in the cellular accumulation and efflux of paclitaxel in NCI/ADR-RES cells (Figure 6C).

### Globo series GSLs modulate *MDR1 *expression

**Figure 7:**
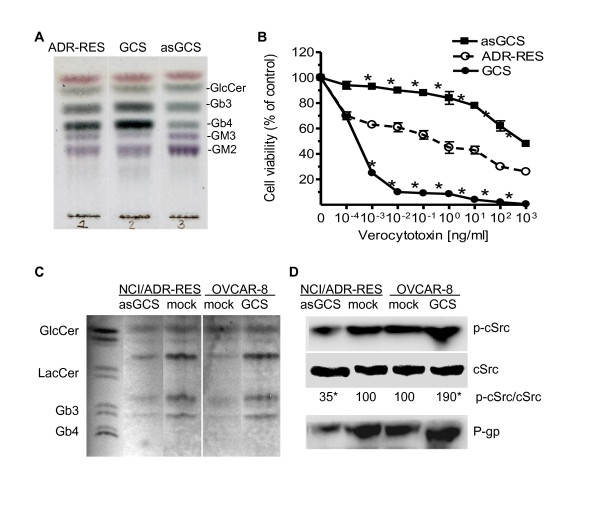
**Globo Series GSLs Mediate *MDR1 *Transactivation**. (A) Glycosphingolipids. Cells were cultured in 10% FBS RPMI-1640 medium and harvested by trypsin-EDTA. Extracted lipids (5 μl aliquot of 100 μl) were resolve by TLC and GSLs were visualized by spraying with diphenylamine-aniline phosphoric acid reagent. GlcCer, glucosylceramide. (B) Gb3, a receptor of verocytotoxin on GCS transfectants. Cells were incubated with increasing concentrations of verocytotoxin in 5% FBS RPMI-1640 medium for 72 hr. *, p < 0.001 compared to ADR-RES. (C) GEM GSLs. GEMs of cells were prepared with gradient sucrose and extracted lipids (100 μg of GEM protein) were applied to HPTLC plates. (D) cSrc phosphorylation in GEMs. Equal amounts of GEM protein (50 μg/lane) were resolved by 4-12% gradient SDS-PAGE and immunoblotted with antibodies. p-cSRc/cSrc represents optical densities of the bands; *, p < 0.001 compared with mock.

It has been reported that glycosphingolipids-enriched microdomains (GEMs) or rafts on cell membranes can mediate cSrc kinase activation [39-41]. To clarify which GSL has a major role in mediating *MDR1 *expression, we analyzed GSL profiles of NCI/ADR-RES variants. It was found that the levels of globo series GSLs including globotriaosylceramide (Gb3) and globotetraosylceramide (Gb4) were significantly increased in NCI/ADR-RES/GCS cells and decreased in NCI/ADR-RES/asGCS cells (Figure 7A). On the other hand, N-acetylneuraminyl-α2,3-galactosyl-β1,4-glucosyl ceramide **(**GM3) and N-acetylgalactosaminyl-β1,4-(α2,3N-acetylneuraminyl) galactosyl-β1,4-glucosyl ceramide (GM2) were increased in NCI/ADR-RES/asGCS cells. As a receptor for verocytotoxin, the levels of Gb3 on cells are associated with the cytotoxicity of verocytotoxin [42-44]. To assess the levels of Gb3 in NCI/ADR-RES variants, we examined cell viability in response to verocytotoxin. As shown in Figure 7B, NCI/ADR-RES/asGCS cells were substantially resistant, while NCI/ADR-RES/GCS cells were extremely sensitive to verocytotoxin. The EC_50 _for verocytotoxin was increased by 4,000-fold (8 × 10^2 ^*vs*. 2 × 10^-1 ^ng/ml) in NCI/ADR-RES/asGCS cells and was decreased by 400-fold (2 × 10^-1 ^*vs*. 5 × 10^-4 ^ng/ml) in NCI/ADR-RES/GCS cells.

Furthermore, we examined whether GCS alter Gb3 concentration and cSrc kinase in GEMs. As shown in Figure 7C and 7D, silencing of GCS by MBO-asGCS (100 nM) significantly decreased Gb3 level, and p-cSrc to 32% in GEMs of NCI/ADR-RES cells. On the contrary, GCS transfection significantly increased Gb3 and doubled p-cSrc in GEMs of OVCAR-8 cells. Alterations of Gb3 and cSrc kinase in GEMs following GCS gene manipulations significantly changed P-gp expression levels as well.

**Figure 8:**
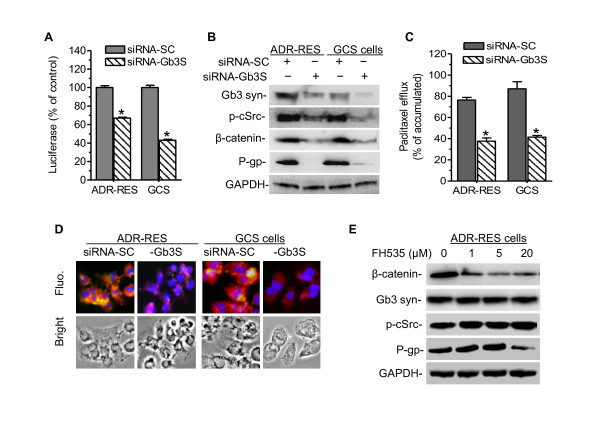
**Gb3 Synthesis and β‐Catenin Recruitment Are Involved in MDR1 Transactivation.** To silence Gb3 synthase, cells were transfected with siRNA-Gb_3_S (100 nM) or control siRNA (siRNA-SC) twice and grown in 10% FBS RPMI-1640 medium for 7 days. (A) *MDR1 *promoter activity. *, P < 0.001 compared with siRNA-SC. (B) Western blot. Gb3 syn, Gb3 synthase; p-cSrc, phosphorylated cSrc. (C) Cellular efflux. *, p < 0.001 compared with siRNA-SC. (D) Immunostaining. Cells were incubated with anti-human Gb3 synthase (red) and anti-P-gp (green) following addition of Alexa 667- and Alexa 488-conjugated secondary antibodies. DAPI in mounting solution was used for nucleus counterstaining (blue). Fluo., merged fluorescence microphotograph (x 200). (E) β-catenin/Tcf4 on P-gp expression. NCI/ADR-RES cells were exposed to FH535, β-catenin/Tcf4 inhibitor in 5% FBS medium for 24 hr.

In order to characterize the role of GSLs in *MDR1 *expression, we selectively silenced the enzyme responsible for the synthesis of globo series GSLs. Cells were transfected with siRNA against Gb3 synthase to block globo series GSL production. As shown in Figure 8A, silencing Gb3 synthase significantly decreased *MDR1 *promoter activity, particularly in NCI/ADR-RES/GCS cells (p < 0.001, compared with NCI/ADR-RES cells). Consistently, silencing of Gb3 synthase considerably decreased p-cSrc, β-catenin and P-gp protein levels and efflux in both cell lines, as detected in Western blot (Figure 8B), cellular efflux (Figure 8C) and immunostaining (Figure 8D). We further treated NCI/ADR-RES cells with FH535, inhibiting β-catenin recruitment to T-cell factor (Tcf) [45]. We found that FH535 (20 μM) decreased P-gp protein to 25% of control (Figure 8E); however, it did not affect either Gb3 synthase or p-cSrc in Western blotting.

## Discussion

GCS is a key enzyme for ceramide glycosylation and GSL synthesis. This study demonstrates that GCS upregulates *MDR1 *expression and modulates drug resistance of cancer. It reveals that GSLs, in particular globo series GSLs mediate gene expression through *cSrc *and β-catenin signaling.

Previous works indicate that GCS and *MDR1 *are co-overexpressed in drug-resistant leukemia [33] and in drug-resistant cancer cells including human ovarian cancer (NCI/ADR-RES), cervical cancer (KB-V1), leukemia (HL-60/VCR), melanoma (MeWo Eto) and colon cancer (SW620/AD) [13,28]. However, it is not clear how GCS or MDR1 affects each other to promote drug-resistance. Suppressing GCS with siRNA or a GCS inhibitor, 1-phenyl-2-decanoylamino-3-morpholino-1-propanol (PDMP), down-regulates the expression and function of P-gp in human breast cancer cells [18]; however, inhibition of GCS by other types of GCS inhibitors (N-butyl-deoxygalactonojirimycin, OGB-1; N-nonyl-deoxygalactonojirimycin, OGB2) did not appear to have any effect on P-gp functional activity in chronic lymphocytic leukemia cells, even though OGB-1 and OGB2 sensitized these cells [46]. P-pg has been proposed as a Golgi glucosylceramide flippase that enhances neutral GSL synthesis, since transfection of *MDR1 *increases globo series GSLs, and inhibition of P-gp with cyclosporine A decreases neutral GSL biosynthesis in cells [32,44,47,48]. To characterize the role of GCS in *MDR1*-GCS co-overexpression, we tested P-gp expression after GCS gene silencing in several different types of cancer cells and in tumors. We have found that silencing of the GCS down-regulates P-gp expression, inhibits its efflux activity, and consequently sensitizes MDR cells including NCI/ADR-RES, A2780-AD, KB-A1, SW620/AD and EMT6/AR1 (Figure 1, 2). Furthermore, suppressing GCS with MBO-asGCS substantially decreases P-gp protein, enhances the accumulation of doxorubicin or paclitaxel, and sensitizes tumors to chemotherapy (Figure 3, 4). We have reported that doxorubicin upregulates GCS expression and results in drug resistance in cells [17]. Herein it has been found that doxorubicin treatment up-regulates GCS expression and importantly, P-gp expression in tumors (Dox *vs*. saline, Figure 3B); MBO-asGCS simultaneously suppresses GCS and *MDR1 *overexpression (MBO-asGCS *vs*. saline, Figure 3B), even under doxorubicin challenge (MBO-asGCS + Dox vs. Dox, Figure 3B). Taken together, these results demonstrate that GCS has a regulatory role in *MDR1 *expression and genesis of drug resistance. Inhibition of GCS appears to be an efficient approach not only to prevent the formation of drug resistance during the course of cancer chemotherapy, but also to reverse drug resistance of cancers.

It has taken time to understand how GSLs generated by GCS modulate gene expression. By introducing GCS into OVCAR-8 cells that express low levels of GCS and P-gp, we have found that GCS consequently upregulates *MDR1 *expression and enhances P-gp efflux through cSrc and β-catenin signaling. Inhibition of Src kinase by PP2 further indicates that GSLs in cell membrane may mediate the phosphorylation of cSrc and of β-catenin that decreases β-catenin levels in the nucleus (Figure 5). This finding has been confirmed by selective silencing of GCS (not Gb3 synthase or GD3 synthase) using MBO-asGCS, in NCI/ADR-RES cells that over express GCS and P-gp (Figure 6). The promoter of the human *MDR1 *contains multiple Tcf4/LEF (T-cell factor 4/lymphoid enhancer factor) binding motifs, CTTTGA/TA/T [49,50]. It has been demonstrated that *MDR1 *is a direct target gene of the β-catenin/Tcf4 transcriptional complex, and activation of β-catenin increases P-gp expression [51-53]. It has been reported that active cSrc elevates the levels of β-catenin, and inhibition of cSrc decreases the binding of β-catenin to the promoters of β-catenin/Tcf4 complex targets such as cyclin D1 and c-Myc [54,55]. In present study, inhibitions of cSrc kinase by PP2 and β-catenin/Tcf4 recruitment by FH535 sequentially prevent MDR1 transactivation (Figure 5, 6, 8). These data strongly support the model that GCS enhances cSrc signaling and β-catenin, and transactivates MDR1 expression.

**Figure 9:**
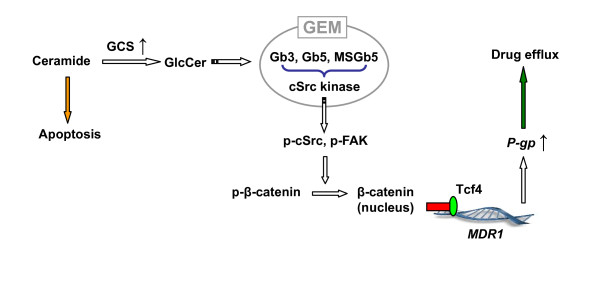
**GSL Synthesis and *MDR1 *Expression**. GCS, glucosylceramide synthase; GlcCer, glucosylceramide; Tcf4, T-cell factor 4; FAK, focal adhesion kinase; cSrc, proto-oncogene (Schmidt-Ruppin A-2); Gb3, globotriaosylceramide; Gb5, globopentaosylceramide; MSGb5, monosyl-Gb5.

GCS is the rate-limiting enzyme in GSL synthesis. Glucosylceramide, the product of GCS, is further converted to lactosylceramide (Galβ1-4GlcCer) by lactosylceramide synthase (glucosylceramide 1,4-galactosyltransferase). Lactosylceramide is the common precursor of nearly all the neutral GSLs and gangliosides. GSL profiling and selective silencing of Gb3 synthase indicate that globo series GSLs (Gb3, Gb4) have more important role than ganglioside in mediating *MDR1 *transactivation and expression (Figure 7, 8). GSLs in either tumor cells or normal cells are clustered and assembled with specific membrane proteins and signal transducers to form GSL-enriched microdomains (GEMs) or rafts [39,56,57]. Gb3 (defined as CD77) is associated with Src family Yes kinase on GEMs [58]. Shiga toxin binds to its receptor Gb3, and activates cSrc kinase [59,60]. Stimulation of monosialyl-Gb5 by its antibody RM1 activates cSrc on GEMs and increases β-catenin in MCF-7 cells [39]. AdamantylGb3, a water-soluble Gb3 mimic significantly increases *MDR1 *expression [61]. Inhibition of GCS by D-PDMP indicates Globo series of GSLs are required for Src kinases that are associated with and concentrated on GEMs [62]. In this study we have found that introduction of GCS or silencing of GCS significantly increases or decreases the levels of Gb3 in entire cells, and particular in GEMs (Figure 7A,B,C). Gb3 levels affect cSrc kinase and result in p-cSrc alterations in GEMs, and consequentially P-gp expression (Figure 7). Taken together, we propose that GCS regulates *MDR1 *expression through activation of cSrc and β-catenin signaling, as depicted in Figure 9. Overexpression of GCS produces large amounts of glucosylceramide when cancer cells are under chemotherapy stress. Enhancement of globo series GSLs (Gb3, Gb5, MSGb5) on the GEMs activates cSrc kinase and β-catenin signaling; nuclear β-catenin with Tcf4 binds to the *MDR1 *promoter and upregulates *MDR1 *expression. Increase of P-gp extrudes anticancer drug out of cells and leads to cancer resistance. Conversely, MBO-asGCS silences GCS gene and down-regulates P-gp through decreasing cSrc and β-catenin signaling.

## Conclusions

This study demonstrates, for the first time, that GCS upregulates *MDR1 *expression and modulates drug resistance of cancer. GSLs, in particular of globo series GSLs mediate gene expression of *MDR1 *through *cSrc *and β-catenin signaling.

## Methods

### Cell culture

Drug-resistant NCI/ADR-RES human ovarian cancer cells (designed as MCF-7-AdrR previously) [63,64] were kindly provided by Dr. Kenneth Cowan (UNMC Eppley Cancer Center, Omaha, NE) and Dr. Merrill Goldsmith (National Cancer Institute, Bethesda, MD, USA). The ovarian carcinoma cells A2780-AD, which is resistant to doxorubicin [65], was kindly provided by Dr. Thomas C. Hamilton (Fox Chase Cancer Center, Philadelphia, PA). Doxorubicin-selected KB-A1 cells [66] were from Dr. Michael M. Gottesman (National Cancer Institute, Bethesda, MD). Drug resistant SW620/Ad colon cancer cells [67] were kindly provided by Drs. Susan Bates and Antonio Fojo (National Cancer Institute, Bethesda, MD). Drug-resistant murine EMT6/AR1 breast carcinoma cells [68,69] were kindly provided by Dr. Ian Tannock (Ontario Cancer Institute, Toronto, ON, Canada). The OVCAR-8 human ovarian carcinoma cells were provided by Dr. M. Hollingshead of Division of Cancer Treatment and Diagnosis Tumor Repository at National Cancer Institute (Frederick, MD). NCI/ADR-RES, KB-A1 and SW620Ad cells were maintained in RPMI-1640 medium containing 10% (v/v) FBS, 100 units/ml penicillin, 100 μg/ml streptomycin, and 584 mg/liter L-glutamine. A2780-AD cells were cultured in RPMI-1640 medium containing 100 nM doxorubicin in addition to the above components. EMT6/AR1 cells were maintained in Dulbecco's modified eagle medium (DMEM) containing 1 μg/ml of doxorubicin for 2 days/week in addition to the above components. Cells were cultured in a humidified incubator with 95% air and 5% CO_2 _at 37°C. Doxorubicin hydrochloride was purchased from Sigma (St. Luis, MO). NCI/ADR-RES cells transfected with human GCS gene (NCI/ADR-RES/GCS) and GCS antisense (NCI/ADR-RES/asGCS) were cultured in RPMI 1640 containing the above components and G418 (400 μg/mL) [13,70].

### Mixed-backbone oligonucleotide and inhibitors

A mixed-backbone oligonucleotide, designed to target the ORF 18-37 of human GCS [34,71], was verified and designated as MBO-asGCS [35]. MBO-asGCS were 20-mer phosphorothioate DNAs, except that four bases at the 5' end and the 3' end were replaced by 2'-O-methyl RNA. MBO-asGCS was synthesized and purified by reverse-phase HPLC and desalting (Integrated DNA Technologies, Inc., Coralville, IA). The MBO-asGCS was introduced into cells with Lipofectamine™ 2000 (Invitrogen, Carlsbad, CA) in Opti-MEM I reduced-serum medium (Invitrogen). To repress *MDR1 *expression, cells were transfected with MBO-asGCS (100 nM) twice and grown in 10% FBS RPMI-1640 medium for 7 days. To inhibit P-gp function, NCI/ADR-RES cells were exposed to verapamil (10 μM) in 5% RPMI-1640 at 37°C for 2 hr, before the analysis of accumulation and efflux. Verapamil hydrochloride was purchased from Sigma-Aldrich (St. Louis, MO).

To silence Gb3 synthase, NCI/ADR-RES and NCI/ADR-RES/GCS cells were transfected with siRNA targeting human Gb3 synthase (siRNA-Gb_3_S 100 nM) or scrambled control siRNA (siRNA-SC 100 nM) twice and grown in 10% FBS RPMI-1640 medium for 7 days. The siRNA targeting human Gb3 synthase and control siRNA-A were purchased from Santa Cruz Biotechnology (Santa Cruz, CA, USA). β-1,3-Gal-TL siRNA (sc-62006) was designed to knockdown human β-1,3-galactosyltransferase (GeneID: 145173). Control siRNA-A was consists of a scrambled sequence that will not lead to the specific degradation of Gb3. siRNAs (100 nM) were introduced into these cells with Lipofectamine 2000.

A Src kinase inhibitor, 4-amino-5-(4-chlorophenyl)-7-(*t*-butyl) pyrazolo[3,4-*d*]pyrimidine (PP2) [37,38] was purchased from Enzo Life Sciences (Plymouth Meeting, PA). An effective β-catenin/Tcf inhibitor, FH535 [45] was purchased from Sigma-Aldrich (St. Louis, MO). OVCAR-8/GCS cells were incubated with PP2 (10 μM) in 5% RPMI-1640 medium for 24 hr. NCI-ADR-RES cells were exposed to FH535 (1 to 20 μM) in 5% RPMI-1640 medium for 24 hr.

### Western blotting analysis

Western blotting was conducted as described previously [13,17]. After treatments, cells or tissue homogenates were lysed using NP40 cell lysis buffer (Biosource, Camarillo, CA, US) to extract the total cellular protein for Western blot. The nuclear proteins were extracted as described previously [72]. Briefly, cells were suspended in 100 μl of Tween-20 lysis buffer (25 mM Tris/Hepes, pH 8.0, 250 mM NaCl, 2 mM EDTA, 1 mM phenylmethylsulfonyl fluoride, 0.5% Tween-20), and kept on ice for 15 min. The nuclei were pelleted at 6000 *g *for 5 min at 4°C, and then resuspended in 100 μl of the lysis buffer containing 500 mM NaCl and incubated on ice for additional 15 min. After incubation, the samples were mixed with 100 μl of the lysis buffer (without NaCl). The supernants were collected for Western blotting following a spin-down at 10,000 *g *for 15 min. Equal amounts of these proteins (50 μg/lane) were resolved using 4-20% gradient SDS-PAGE (Invitrogen). The transferred blot was blocked with 5% fat-free milk in PBS and immuno-blotted with primary antibodies (anti-GCS goat IgG, anti-P-pg mouse, cSrc, phosphorylated cSrc, phosphorylated FAK, β-catenin, phosphorylated β-catenin) at 4°C, overnight. The antigen-antibody in blots was detected by using a second antibody-conjugated HRP and enzyme-linked chemiluminescence plus substrate (GE Healthcare). GAPDH or β-tubulin was used as loading control for total proteins or nuclear proteins.

### Immunohistochemistry

Cells (10,000 cells/chamber) were grown in 4-chamber slides with 10% FBS culture medium for 48 hr. After methanol fixation, cells were blocked and then incubated with anti-GCS serum and anti-P-gp antibody (1:100) in block solution (Vector Laboratories, Burlingame, CA), overnight at 4°C. GCS antibody and P-gp antibody on cells were recognized by Alexa Fluor^®^488 goat anti-rabbit IgG and Alexa Fluor 667 goat anti-mouse IgG (Invitrogen). Cell nuclei were counterstained with DAPI (4', 6 diamidino-2-phenylindole) in mounting solution (Vector Laboratories). The slides were observed using a Nikon TE-2000 phase contrast microscope, and the images were captured by a Retiga 2300™ monochrome digital camera using IPLab™ image analysis program (Scanalytics Inc., Rockville, MD).

#### Cell viability assay

Cell viability was analyzed by quantitation of ATP, an indicator of active cells using CellTiter-Glo luminescent cell viability assay (Promega, Madison, WI), as described previously [17]. Briefly, cells (4,000 cells/well) were grown in 96-well plates with 10% FBS RPMI-1640 medium for 24 hr. MBO-asGCS (50 nM) was introduced into cells by Lipofectamine 2000 (vehicle) in Opti-MEM reduced-serum medium, for 4 hr. Cells were then incubated with increasing concentrations of agents in 5% FBS medium for another 72 hr. Cell viability was determined by the measurement of luminescent ATP using a Synergy HT microplate reader (BioTek, Winnooski, VT. USA), following incubation with CellTiter-Glo reagent (Promega, Madison, WI, USA).

Verocytotoxin was kindly provided by Dr. Clifford A. Lingwood (University of Toronto and Hospital for Sick Children, Toronto, Canada). After 24 hr growth in 96-well plates, cells were incubated with verocytotoxin in 5% FBS RPMI-1640 medium for an additional 72 hr.

### Cellular ceramide glycosylation assay

Cells were grown 24 hr in 35-mm dishes (1 × 10^6 ^cells/dish) in 10% FBS RPMI-1640 medium and MBO-asGCS (50 nM) was introduced as described above. After 12 hr growth in 10% RPMI-1640 medium, cells were switched to 1% bovine serum albumin (fatty acid free) medium containing 50 μM NBD C_6_-ceramide complexed to BSA (Invitrogen). After 2 hr incubation at 37°C, lipids were extracted, and resolved on partisil high performance TLC plates with fluorescent indicator in a solvent system containing chloroform/methanol/3.5 N ammonium hydroxide (85:15:1, v/v/v), as described previously [17,73]. NBD C_6_-glucosylceramide and NBD C_6_-ceramide were identified using AlphaImager HP imaging system (Alpha Innotech, San Leandro, CA) and quantitated on a Synergy HT multi-detection microplate reader (BioTek). For quantitation, calibration curves were established after TLC separation of NBD C_6_-ceramide (Invitrogen) and NBD C_6_-glucosylceramide (*N*-hexanol-NBD-glucosylceramide; Matreya, Pleasant Gap, PA).

### Glycosphingolipid analysis

Cells were cultured in 10% FBS RPMI-1640 medium and harvested by trypsin-EDTA. Approximately 400 mg of pelleted cells was lyophilized and extracted twice with 4 ml of chloroform/methanol (2/1, v/v). The two extracts were combined, evaporated to dryness and subjected to saponification by suspending the residue in 1 ml of 0.5 N NaOH. After incubation at 55°C for 1 hr, the mixture was neutralized with glacial acetic acid, evaporated to dryness, suspended in 1 ml of water, exhaustively dialyzed against water and lyophilized. The lyophilized powder was dissolved in 100 μl chloroform/methanol (2/1) and a 5-μl aliquot was spotted on a TLC plate (Merck, Darmstadt, Germany). The plate was developed in chloroform/methanol/12 mM MgCl_2 _(50/40/10, v/v/v), and GSLs were visualized by spraying the plate with diphenylamine-aniline phosphoric acid reagent as described previously [74].

GSLs on GEMs were prepared and analyzed in NCI/ADR-RES/asGCS, OVCAR-8/GCS and each mock-transfected cell lines, as described previously [58,75] with modification. Briefly, cells (1 × 10^7^) were harvested, suspended in 1 ml of lysis buffer containing 1% Triton X-100 (TX-100), and 75 units of Aprotinin in TNEV solution (10 mM Tris-HCl, pH 7.5, 150 mM NaCl, 5 mM EDTA, 1 mM NaVO_4_), homogenized and incubated on ice for 20 min. Cell lysates were centrifuged for 5 min at 1300 *g *to remove nuclei and large cellular debris. The supernatant collected (700 μl) was mixed with equal volume (700 μl) of 85% sucrose (wt/vol) in TNEV solution. The diluted Triton X-100 lysates were overlaid with 30% (6 ml) and 5% (3.3 ml) of sucrose TNEV solution in SW41 centrifuge tube. The samples were centrifuged for 18 h at 200,000 *g *at 4°C. White bands located at ~5-7% sucrose were collected as GEM fraction and its protein content was determined using BCA Protein Assay Kit. The lipids were extracted with chlofrom/methanol/water (1:1:1, v/v/v) from 200 μg of GEM protein. Extracted lipids were resuspended in choloform-methanol (1:1, v/v) and applied to partisil HPTLC plates. Lipids were resolved using the solvent system of chloroform/methanol/water (65:25:4 v/v/v). Acid alcohol (90% methanol/5% sulfuric acid, 5% acetic acid; Sigma-Aldrich) was used for the chemical detection of glycosphingolipids. Neurtral glycospingolipids qualmix and ceramide trihexosides (Gb3) were purchased from Matreya (Pleasant Gap, PA) and used as standards in TLC.

### High-pressure liquid chromatography (HPLC) analysis of doxorubicin

The concentrations of doxorubicin in cells, serum and tumors were analyzed, as described previously with minor modifications [76,77]. Cells (2.5 × 10^5 ^cells/well) were grown in 6-well plates with 10% FBS RPMI-1640 medium. After 24 hr, cells were shifted to medium containing doxorubicin (100 μM) for 2 hr incubation, at 37°C. Following ice-cold PBS rinsing, cellular doxorubicin was extracted using 3 ml of methanol. For tumor samples, ~80 mg of tissue was homogenized in 200 μl of ice-cold methanol. After centrifugation (7,000 *g*, 10 min), the supernatant of samples was injected into the HPLC system with an auto-sampler. Doxorubicin was resolved on a Pecosphere C18 reversed-phase column with mobile phase of 50 mM sodium phosphate buffer (pH 2.0):acetonitrile:1-propanol (65:25:2; v/v/v; flow rate of 0.8 ml/min). Doxorubicin was detected with the use of a scanning fluorescence detector at λ_excitation _480 nm and λ_emission _550 nm. The retention time was approximately 7 minutes for doxorubicin. Standard curves were linear within the range of 1 ng/ml to 100 ng/ml (equal to 0.002 ~ 0.17 μM). Samples containing high doxorubicin concentrations were diluted as needed.

For the analysis of doxorubicin in serum, proteins were precipitated with 10% trichloroacetic acid. The supernatant obtained after centrifugation (7,000 *g*, 10 min) was used for HPLC assay.

### Paclitaxel accumulation and efflux

The measurements were performed as described previously [78,79]. After treatments or transfection, cells were grown in 10% FBS RPMI-1640 medium for 24 hr and then shifted to 5% FBS RPMI-1640 medium containing Fluotax-2 (Oregon green 488 paclitaxel, 0.5 μM) and incubated at 37°C for 2 hr. After ice-cold wash and trypsinization, accumulation of paclitaxel was measured. For efflux, at the end of the 2 hr incubation, fresh media was added following wash and re-incubated at 37°C for an additional 2 hr. Fluorescent paclitaxel was measured at λ_excitation _485 nm and λ_emission _529 nm using a Synergy HT microplate reader. Cellular accumulation of paclitaxel was normalized to cell number and paclitaxel added (total intensity). The efflux was normalized against accumulated paclitaxel in cells. Flutax-2 (Oregon green 488 paclitaxel) was purchased from Invitrogen.

After two MBO-asGCS administrations (1 mg/kg every 3-days, ip, 3 mice/group), the small intestine (ileum) and tumors were resected. Tissues (25 mg/reaction) were incubated with fluorescent paclitaxel (1.0 μM) in 200 μl of 1% BSA RPMI-1640 medium containing collagenase IV, immediately following mincing. Accumulation of paclitaxel was measured after 2 hr incubation and 3 times of washes with ice-cold PBS. For efflux, samples were incubated with fresh medium for an additional 2 hr following accumulation and washed 3 times with ice-cold PBS.

### Drug-resistant tumor models and treatments

Drug-resistant NCI/ADR-RES tumors were established by using the methods described previously [35,80]. Athymic nude mice (*Foxn1*^*nu*^/*Foxn1*^*+*^, 4-5 weeks, female) were purchased from Harlan (Indianapolis, IN) and maintained in the Vivarium, University of Louisiana at Monroe, according to the approved protocol. Cultured cells after 3 to 5 passages were washed with and resuspended in serum-free RPMI-1640 medium. A suspension of NCI/ADR-RES cells (1 × 10^6 ^cells in 20 μl per mouse) was injected into the left flank of the mouse. The mice were monitored by measuring tumor growth, body weight and clinical observation. Tumor-bearing mice were randomly divided into multiple treatment and control groups (ten mice per group). MBOs, dissolved in RPMI 1640 medium were given at the dose of 1 mg/kg, twice per week, at the tumor site. The control group received medium only. In combination therapy, doxorubicin was given by intraperitoneal injection at 2 mg/kg once a week with medium or MBOs for 42 days, respectively.

Tumors were removed, fixed and maintained in paraffin blocks. Microsections from each tumor (5 μm) were H&E stained and identified by pathologist (Dr. J. Bao). For immunostaining, antigens were retrieved in steaming sodium citrate buffer (10 mM, 0.05% Tween-20, pH 6.0, 10 min). After blocking with 2% block solution (Vector Laboratories, Burlingame, CA), the slides were incubated with primary antibodies (1:100) at 4°C, overnight.

### *MDR1 *promoter assay

The human *MDR1 *promoter reporter, pMDR1 [81] was kindly provided by Dr. Kathleen W. Scotto (University of Medicine and Dentistry of New Jersey, New Brunswick, NJ). *MDR1 *promoter (sequence from -1202 to +118) drives luciferase expression from pGL2B. After treatments or transfection, cells (2.5 × 10^5 ^cells/well) were placed into 6-well plates with 10% FBS RPMI-1640 medium. After 24 hr culture, pMDR1 plasmid (4 μg/well) and pGL4 *renilla *luciferase reporter driven by thymidine kinase promoter (pGL4-hRluc/TK; 4 μg/well) were introduced into cells with Lipofectamine 2000 and cells were cultured in 10% FBS medium for additional 48 hr. Cell lysates were incubated with Dual-luciferase reporter assay system reagents (Promega). The intensities of *firefly *luciferase (*MDR1 *promoter activity) and *renilla *luciferase (TK promoter activity) were measured using a Synergy HT multidetection microplate reader. *MDR1 *promoter activity was normalized to protein and TK promoter.

### Statistic analysis

All data represent the mean ± SD. Experiments in triplicate were repeated 2 or 3 times in cell models. Student's *t *test was used to compare mean values, using a Prism 4 program (GraphPad software, San Diego, CA).

## Competing interests

The authors declare that they have no competing interests.

## Authors' contributions

YYL conceived, designed the experiments and carried out parts of Western blot, immunostaining, the generation of GCS transfectants, animal study and verocytotoxin assays; YYL further performed all data analysis, writing of the manuscript, including preparations of all figures.

VG carried out HPLC analysis of doxorubicin, ceramide glycosylation, Gb3 in GEMs, promoter activity assay, nuclear protein preparation, cell culture and GCS transfection. VG participated in data analysis, figure preparation and critical reading of the draft.

GAP carried out Western blot analysis, cell culture, animal study and P-gp efflux assay. GAP participated in data analysis, figure preparation and critical reading of the draft.

KB carried out plasmid DNA preparation and parts of promoter activity assay.

YZ carried out immunostaining and critical reading of the draft.

JB carried out tissue section preparation, H&E staining and tumor characterization.

HM carried out parts of HPLC analysis of doxorubicin and critical reading of the draft.

MCC participated in parts of experimental design and critical reading of the draft.

YTL carried out glycosphingolipid assay and critical reading of the draft.

SMJ participated in experimental design and critical reading of the draft.

All authors read and approved the final manuscript.
